# Patellar Tendon Repair With Gracilis Autograft Augmentation and Anchor Fixation

**DOI:** 10.1002/atn2.70145

**Published:** 2026-05-28

**Authors:** Luis Henrique Longo, Bruno Dada Gulini, Marcos Paulo Tercziany Vanzin, Fernando Martins Rosa, Luca Eiji Sohn Sato, Luis Antonio de Ridder Bauer, Edmar Stieven Filho

**Affiliations:** ^1^ Knee Surgery Group, Department of Orthopaedic Surgery Complexo Hospitalar do Trabalhador Curitiba Brazil; ^2^ Hospital de Clínicas da Universidade Federal do Paraná Curitiba Brazil

## Abstract

Patellar tendon rupture is a severe injury that disrupts knee extensor continuity and requires prompt surgical management. Although primary repair remains the standard treatment, isolated suture repair may be insufficient in cases of poor tissue quality or high mechanical demand, making augmentation necessary. Autologous hamstring grafts provide reliable biological reinforcement, and the gracilis tendon offers favorable handling characteristics, adequate tensile strength when doubled, and low donor‐site morbidity. Hamstring grafts also show higher load‐to‐failure values than the native tendon, supporting their use to enhance initial construct stability. This Technical Note describes a reproducible technique for patellar tendon repair augmented with a doubled gracilis autograft, using suture‐anchor fixation exclusively, without the need for patellar transosseous tunnels. The graft is positioned along the medial and lateral borders of the tendon to reinforce the primary repair while preserving patellar integrity. This approach offers a mechanically robust and biologically supported construct, providing an alternative to traditional methods in cases where additional reinforcement is warranted.

## Graphical abstract

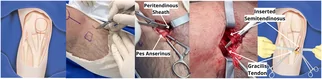

**VIDEO 1:** Step‐by‐step demonstration of the surgical technique for patellar tendon repair with doubled gracilis autograft augmentation and suture‐anchor fixation. The video illustrates patient positioning, graft harvesting, patellar preparation, anchor placement, primary tendon repair, graft passage, and final tibial fixation. Video content can be viewed at https://doi.org/10.1002/atn2.70145.

Patellar tendon rupture is a severe injury requiring surgical intervention to restore the knee extensor mechanism.^1^, ^2^ Although primary suture repair is the standard, it may be insufficient in cases of poor tissue quality or high functional demand, leading to a risk of gap formation or rerupture.^3^, ^4^ Biological augmentation with an autologous hamstring graft can enhance the mechanical strength and biological environment of the repair.^5^, ^6^

This Technical Note describes a reproducible technique for patellar tendon repair augmented with a doubled gracilis autograft. This approach is distinguished by 2 key aspects: first, the exclusive use of suture anchors for proximal graft and tendon fixation, which preserves patellar bone stock and avoids the iatrogenic fracture risk associated with transosseous tunnels;^7^ and second, the use of an isolated gracilis tendon, which serves as a simple, effective, and low‐morbidity biological reinforcement.^8^, ^9^

## SURGICAL TECHNIQUE

A detailed demonstration of the technique is provided in Video 1. Key steps, pearls, and pitfalls are summarized in Table 1.

**TABLE 1:** Step‐by‐Step Guide and Surgical Pearls

Step‐by‐Step Guide	Surgical Pearls	Pitfalls
1. Supine positioning with gluteal bump; tourniquet applied but not routinely inflated	Maintain patella at zenith and limb in neutral rotation	Inadequate exposure can lead to malpositioned incisions or nerve injury
2. Longitudinal patellar tendon incision (8 cm) and pes anserinus incision (1 cm distal, 2 cm medial to tibial tuberosity)	Premark incisions for accurate graft passage	Failure to premark can result in incision malposition and cosmetic or functional deficits
3. Identify and release gracilis subperiosteally; harvest with closed stripper	Protect saphenous nerve. Keep stripper aligned and under gentle traction	Aggressive stripping can amputate the graft; inadequate release can damage the saphenous nerve
4. Prepare graft: clean, measure (16‐18 cm), double to form two‐strand construct; whipstitch both ends with No. 2 suture	Doubled gracilis yields 28 to 32 mm^2^ cross‐section and 400 to 500 N load‐to‐failure^8^, ^9^	Insufficient graft length may compromise the doubled construct
5. Expose rupture, remove hematoma/devitalized tissue, mobilize stump	Refresh tendon edges and assess tissue quality	Over‐debridement weakens the native tendon; under‐debridement risks poor healing
6. Expose inferior patellar pole; create shallow trough (3‐4 mm × 10‐12 mm); insert two 5.0‐mm anchors 1.5 to 2 cm apart	Slightly oblique trajectory increases pullout strength; maintain drill depth 15 to 18 mm	Anchor pullout if trajectory is too perpendicular; patellar fracture if too deep
7. Fix graft midpoint to anchors using 4 to 5 square knots in crossed configuration	Confirm equal limb length and avoid knot bulk	Asymmetric tensioning can lead to graft maltracking or failure
8. Perform primary tendon repair with double Krackow sutures using anchor threads	Avoid overtensioning; verify patellar height versus contralateral; tension with knee in full extension	Overtensioning can cause patella baja; undertensioning risks persistent instability
9. Retrieve medial limb through pes incision; drill transverse tibial tunnel (4.5‐5.0 mm, 1 cm distal/posterior to tibial tuberosity)	Drill at 30° flexion to avoid anterior cortex breach. Match tunnel to graft diameter	Tunnel misplacement can compromise fixation or cause iatrogenic fracture
10. Pass both limbs through the tunnel in opposite directions using shuttle suture	Crossing limbs improves load distribution and anterior reinforcement	Failure to cross limbs can lead to asymmetric load sharing
11. Fix limbs with interference screw (7‐9 mm × 20‐25 mm) from medial side under uniform traction	Match screw to graft/tunnel; avoid anterior cortical prominence	Screw divergence or graft damage during insertion; prominent screw head
12. Assess construct stability and patellar height	Confirm no gapping >2 to 3 mm through ROM	Persistent gapping indicates repair failure and requires immediate re‐evaluation
13. Irrigation, hemostasis, layered closure	Layered closure preserves glide and reduces adhesions	Inadequate closure can lead to wound complications or adhesions

### Patient Positioning and Graft Harvest

**FIGURE 1:**
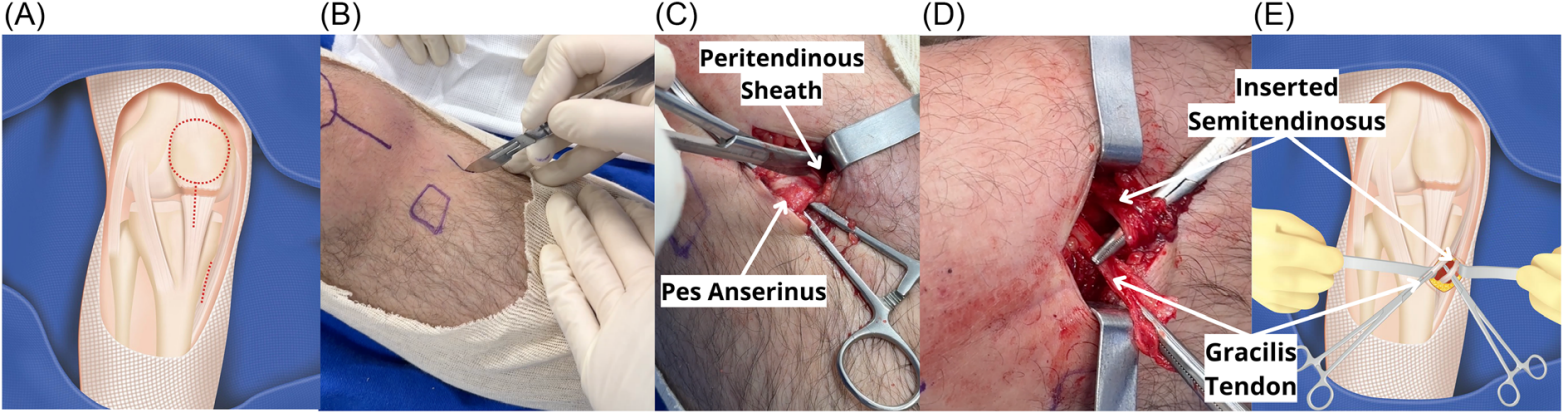
Surgical setup and gracilis tendon harvest in the right knee. An illustrative schematic shows the anatomical landmarks and planned pes anserinus incision (A). The skin incision is made approximately 1 cm distal and 2 cm medial to the tibial tubercle with the limb prepped and draped in the usual sterile fashion (B). Dissection through the sartorial fascia exposes the pes anserinus and the peritendinous sheath (C). The gracilis tendon is identified proximal and slightly superficial to the semitendinosus, which remains inserted during harvest (D). An illustrative schematic shows isolation and harvest of the gracilis graft while preserving the semitendinosus insertion (E).

The patient is placed in the supine position, and a high‐thigh tourniquet is applied but not routinely inflated. Through a 2 cm incision over the pes anserinus, the gracilis tendon is identified, released from its tibial insertion, and harvested using a closed tendon stripper, protecting the saphenous nerve (Figure 1). The graft is prepared by whipstitching both ends with No. 2 nonabsorbable braided polyester sutures (Ethibond, Ethicon, Somerville, NJ, USA). A typical 16 to 18 cm graft is doubled to create a robust 2‐strand construct.

### Tendon Exposure and Patellar Preparation

**FIGURE 2:**
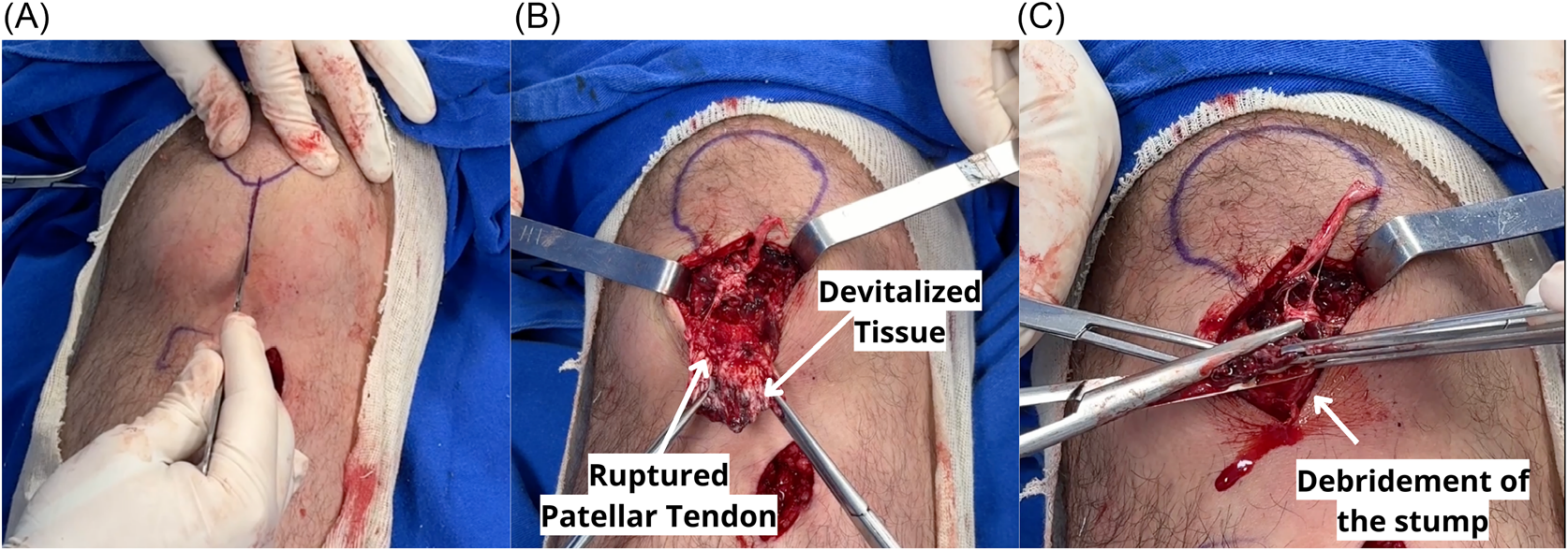
Exposure of the ruptured patellar tendon in the right knee. (A) Longitudinal incision and initial exposure of the rupture site. (B) Identification of devitalized tissue at the proximal stump. (C) Debridement of the tendon edges to prepare a viable surface for reinsertion.

**FIGURE 3:**
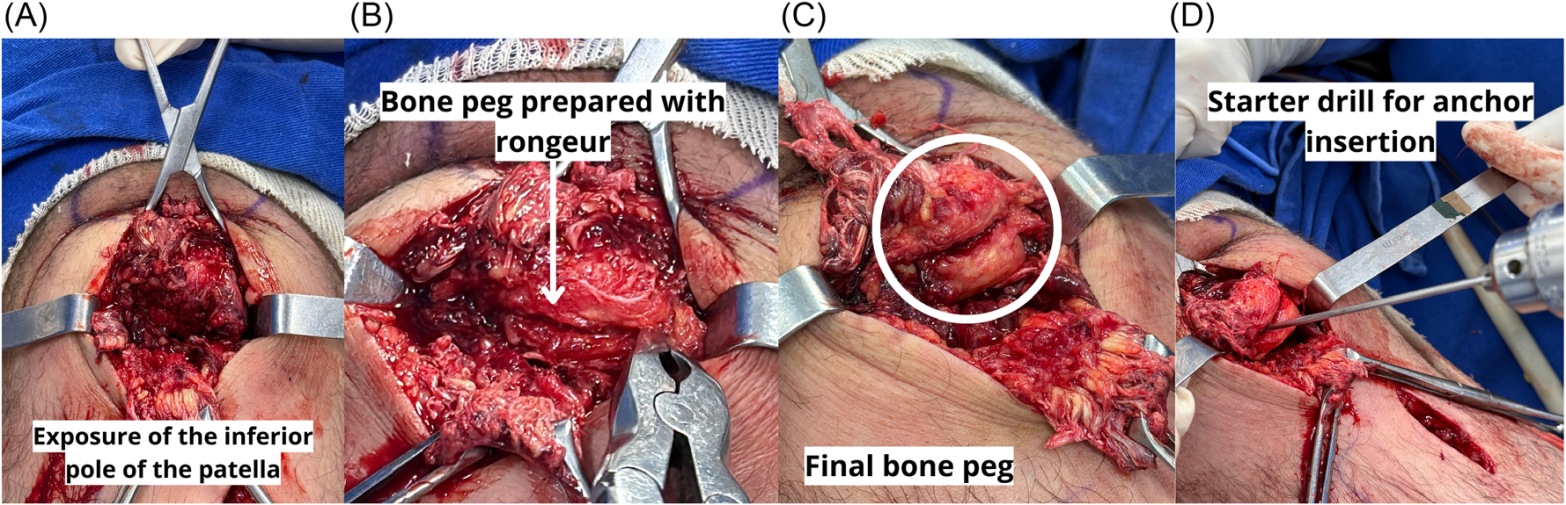
Preparation of the inferior patellar pole in the right knee. (A) Exposure of the distal patella after debridement of the bone‐tendon interface. (B) Creation of a central bone trough using a rongeur. (C) Final view of the prepared bone peg. (D) Drilling of pilot holes for 5.0‐mm suture anchor insertion.

A longitudinal incision is made to expose the patellar tendon rupture. The paratenon is preserved, and the rupture site is debrided of nonviable tissue (Figure 2). The inferior patellar pole is exposed, and a shallow bony trough is created with a rongeur to enhance contact for healing. Two pilot holes are drilled 1.5 to 2 cm apart for 5.0 mm double‐loaded suture anchors (SPINFIX, Setormed, São Carlos, SP, Brazil). The holes are aimed slightly obliquely toward the patellar center to maximize pullout resistance (Figure 3).

### Proximal Fixation and Primary Repair

**FIGURE 4:**
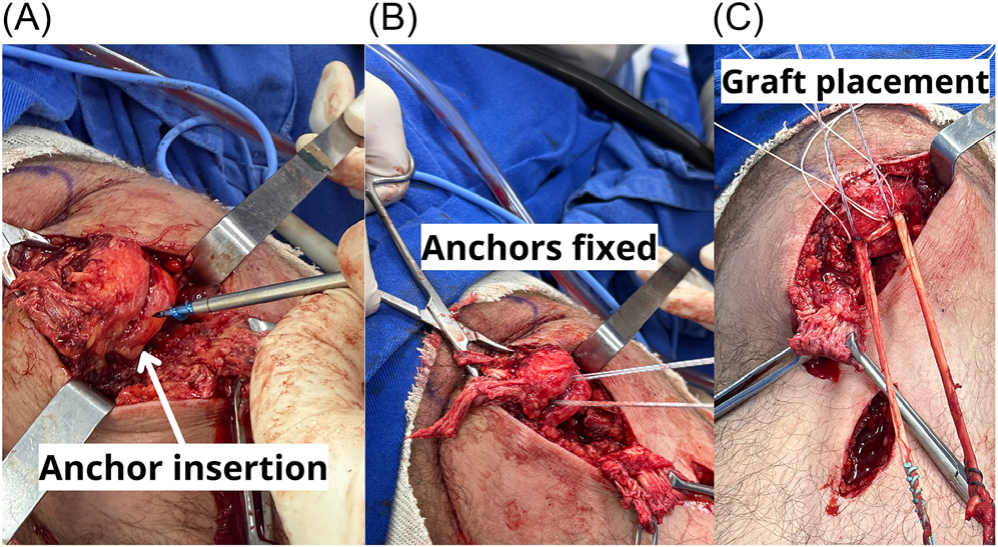
Anchor placement and proximal graft fixation in the right knee. (A) Insertion of two 5.0‐mm double‐loaded suture anchors into the inferior patellar pole. (B) Anchors fixed. (C) Secure fixation of the graft with high‐strength sutures in a crossed configuration, forming the proximal augmentation construct.

**FIGURE 5:**
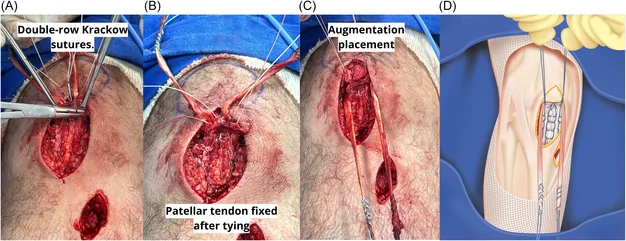
Primary tendon repair and gracilis graft augmentation in the right knee. Double Krackow sutures are placed along the proximal patellar tendon stump using high‐strength sutures from the patellar anchors (A). The tendon is then reduced to the inferior pole of the patella and secured with final knot tying in full knee extension (B). The medial and lateral limbs of the gracilis graft are positioned along the borders of the patellar tendon to form the augmentation construct, as shown in the clinical intraoperative image (C). An illustrative schematic shows the final configuration of the augmentation construct and graft orientation (D).

The 2 suture anchors are inserted into the patella. The central portion of the doubled gracilis graft is secured into the bony trough using the anchor sutures in a crossed configuration (Figure 4). The remaining sutures from the anchors are then used to perform the primary tendon repair. A double Krackow stitch is placed in the proximal tendon stump. With the knee held in full extension to prevent overtensioning, the tendon is reduced to its anatomic footprint and the sutures are tied, ensuring patellar height is restored (Figure 5).

### Distal Graft Passage and Final Fixation

**FIGURE 6:**
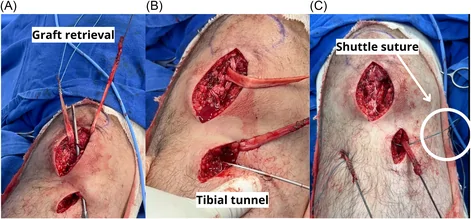
Distal graft passage in the right knee. (A) Retrieval of the medial limb of the gracilis graft through the pes anserinus incision using a grasper. (B) Creation of the transverse tibial tunnel approximately 1 cm distal and 1 cm posterior to the tibial tubercle. (C) Shuttle suture passed through the tibial tunnel to assist in crossing both graft limbs for symmetrical reinforcement of the patellar tendon.

**FIGURE 7:**
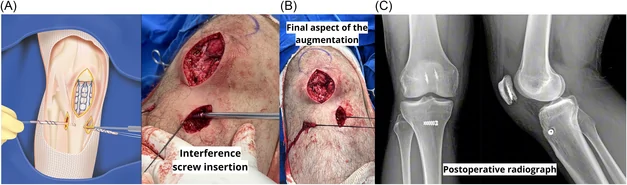
Final fixation and postoperative radiographic assessment of the right knee. An illustrative schematic shows fixation of both gracilis graft limbs using an appropriately sized interference screw inserted through the lateral tibial tunnel opening (A). The final intraoperative image shows the completed augmentation construct, with the reinforced patellar tendon and proper graft orientation (B). Immediate postoperative anteroposterior and lateral radiographs confirm adequate implant positioning and restoration of patellar height and extensor mechanism alignment (C).

The 2 free limbs of the gracilis graft are passed distally along the borders of the repaired tendon. A transverse tibial tunnel (4.5‐5.0 mm) is drilled approximately 1 cm distal and 1 cm posterior to the center of the tibial tubercle, oriented from medial to lateral. This posterior placement helps avoid stress concentration at the tibial crest. The graft limbs are passed in opposite directions through the tunnel (Figure 6). While maintaining firm, symmetric manual tension on both limbs with the knee at 30° of flexion, the construct is secured with a 7‐mm × 20‐mm interference screw (Titanflex, OCX, São Paulo, SP, Brazil) inserted into the tunnel. Final stability is confirmed through a full range of motion (Figure 7).

### Postoperative Rehabilitation

The knee is immobilized in a hinged brace locked in full extension for 2 weeks, with partial weight‐bearing as tolerated. Passive range of motion is initiated at week 2, progressing to 90° of flexion by week 4. Active motion and full weight‐bearing begin at 6 weeks. Return to sport‐specific activities is typically permitted at 4 to 5 months.

## DISCUSSION

This technique provides a mechanically robust and biologically augmented repair for patellar tendon ruptures while mitigating risks associated with traditional methods. The primary advantage is the use of suture anchors for proximal fixation, which avoids the need for transpatellar tunnels and reduces the risk of iatrogenic patellar fracture.^7^ This bone‐preserving approach is biomechanically comparable to tunnel techniques for achieving secure tendon‐to‐bone apposition.

Furthermore, the use of an isolated, doubled gracilis autograft offers reliable biological reinforcement with minimal donor‐site morbidity compared to harvesting both hamstring tendons.^8^, ^9^ The graft enhances the initial strength of the repair, protecting it from gap formation during early rehabilitation.^6^ Key limitations include the need for meticulous surgical technique to ensure correct anchor placement and graft tensioning to restore normal patellar height without overtightening. In conclusion, this anchor‐based augmentation technique is a reproducible and effective option for reinforcing primary patellar tendon repairs.

The main advantages, limitations, and technical considerations of this method are summarized in Table 2.

**TABLE 2:** Advantages and Disadvantages

Advantages	Disadvantages
The use of an isolated gracilis autograft provides biological reinforcement while minimizing donor‐site morbidity compared with combined ST‐G harvest	Potential iatrogenic injury during gracilis tendon harvest or anchor placement
Suture anchor fixation eliminates the need for transpatellar tunnels, reducing the risk of patellar fracture	Requires precise anchor placement to avoid cortical breach or malposition
The gracilis graft offers immediate mechanical support, enhancing early rehabilitation potential	Limited graft diameter may restrict use in cases of extensive tendon loss
Biological augmentation improves tendon healing and reduces the risk of rerupture	Technique demands meticulous tensioning to avoid overtightening or graft laxity
The procedure is reproducible and does not require specialized implants beyond anchors and interference screw	Slightly longer operative time compared to standard primary repair

## DISCLOSURES

The authors (L.H.L., B.D.G., M.P.T.V., F.M.R., L.E.S.S., L.A.R.B., E.S.F.) declare that they have no known competing financial interests or personal relationships that could have appeared to influence the work reported in this paper.
